# Upcycling Shellfish Waste: Distribution of Amino Acids, Minerals, and Carotenoids in Body Parts of North Atlantic Crab and Shrimp

**DOI:** 10.3390/foods13172700

**Published:** 2024-08-27

**Authors:** Abul Hossain, Fereidoon Shahidi

**Affiliations:** Department of Biochemistry, Memorial University of Newfoundland, St. John’s, NL A1C 5S7, Canada; abulh@mun.ca

**Keywords:** *Pandalus borealis*, *Chionoecetes opilio*, shell waste, mineral, astaxanthin, carotenoid

## Abstract

The snow/pink crab (*Chionoecetes opilio*) and Northern shrimp (*Pandalus borealis*) are widely distributed in the North Atlantic Ocean. During processing/consumption, about 80% of the harvest is discarded as processing waste, which is a rich source of protein, chitin, minerals, and carotenoids. This study, for the first time, investigated the proximate composition and individual amino acids, minerals, and carotenoids from different body parts (carapace, shoulder, claw, tip, and leg) of snow crabs and shrimp shells. Shrimp proteins were found to be abundant and well-balanced in their amino acid composition. Compared to shrimp shells, a lower content of amino acids was found in the snow crab, depending on the part of the shell used. Moreover, crab shells, mainly crab claws, contained a higher (*p* < 0.05) level of chitin compared to shrimp shells. Seven micro-elements (Mn, Fe, Cu, Zn, As, Ba, and Ce) and six macro-elements (Ca, Na, K, Mg, P, and Sr) were identified using inductively coupled plasma-mass spectrometry (ICP-MS). Among them, calcium and iron were higher in crab carapaces (*p* < 0.05), followed by shrimp shells and other crab shell segments. Additionally, shrimp and crab carapaces contained a significant level of carotenoids, and these were mainly composed of astaxanthin and its mono- and diesters, along with zeaxanthin, astacene, canthaxanthin, and lutein. Thus, this investigation provides detailed information to allow upcycling of shellfish waste and addresses the knowledge gap concerning the availability of various nutrients in different crab sections and shrimp shells.

## 1. Introduction

The production volume of seafood from wild fisheries and aquaculture is increasing to meet the growing demand for aquatic products. These processing industries produce significant amounts of shell wastes, which have traditionally been hauled into the ocean or dumped into land, causing environmental pollution and impacts thereof. These can be repurposed into new products or utilized to recover components that yield high-value compounds. The shell wastes generated from the shellfish processing industry are rich in high-value compounds, such as polysaccharides (chitin/chitosan), proteins (amino acids/peptides/collagens/enzymes), lipids (polyunsaturated fatty acids), pigments (astaxanthin), as well as micro- and macro-elements [1]. Annually, around 15 million tons of crustaceans are harvested globally; however, the production yield of crustacean meat is relatively low, ranging from 20 to 25%, which results in up to 80% of the raw material being discarded as waste [2].

Northern Shrimp (*Pandalus borealis*) is found in the Northwest Atlantic, ranging from Baffin Bay down to the Gulf of Maine. Northern shrimp generate up to 50% of byproducts or waste, such as heads, shells, and tails, which are a rich source of proteins, minerals, and chitin [3]. Shrimp shells comprise about 25% of the dry mass (dm) and contain 17–20% chitin, 33–40% protein, 34% ash, and 0.3–0.5% lipid [4]. Despite significant efforts, shrimp shells have not been widely utilized. Some have been used as feed additives and protein meals or processed to produce chitin, chitosan, and glucosamine. Consequently, there is a growing interest in using these shells in the preparation of value-added products with desirable functional characteristics. On the other hand, the Atlantic variety of snow crab (*Chionoecetes opilio*) is found in the northeastern Atlantic, ranging from the west coast of Greenland to the Gulf of Maine. Snow crabs have a spider-like shape with an almost circular body and five pairs of legs. Crabs rely on a hard external shell, known as an exoskeleton, for skeletal support and protection. Snow crab is mainly processed as Individually Quick Frozen (IQF) cooked sections, generating up to 30% of waste, containing mainly carapace, viscera, residual meat, hemolymph, and gills. However, crab shells (e.g., carapace, shoulder, claw, tip, and leg) account for up to 80% of the original materials after removing meat. Snow crab processing wastes are presently not commercially utilized in Atlantic Canada, although there is potential to recover them from processing plants as a byproduct. These wastes could be converted into value-added products such as crab meals, lipids, proteins, minerals, chitin, and carotenoids or converted into higher-value bio-products, including peptides, chitosan, omega-3 fatty acids, marine calcium, and astaxanthin [5,6].

Seafood carotenoids are powerful antioxidants that are purportedly stronger than those of vitamins E and C. In particular, astaxanthin is sought after for its numerous health and therapeutic benefits. It is being considered for potential use as a natural food and feed colorant, contributing to its high demand as a metabolite [2,3]. For example, shellfish carotenoids, mainly astaxanthin, have been used in the feed formulation of salmonoid (e.g., salmon, rainbow trout, and Arctic char) for their pigmentation, resulting in improved nutritional value as well as higher market demand due to better consumer acceptance [7]. Aside from this, the most easily exploited sources of chitin are the exoskeleton of crustaceans, mainly crabs and shrimps. Crustacean chitin is extracted primarily from the shells discarded during seafood processing, which have a myriad of applications in biomedical, biodegradable packaging, food and beverage, pharmaceutical, cosmetic, and agricultural fields, as well as environmental remediation, among others [1,8]. Additionally, crustaceans also contain essential minerals, including copper (Cu), zinc (Zn), magnesium (Mg), calcium (Ca), and phosphorus (P), that contribute to overall health and well-being [9]. Apart from playing crucial roles in various physiological processes in the human body, these minerals are important sources of biofertilizers in enhancing soil fertility and promoting plant growth [10]. Furthermore, shellfish are rich in essential amino acids, which are required for numerous physiological processes in crustaceans, including growth, molting, immune response, energy production, nervous system function, reproduction, osmoregulation, and detoxification.

Nevertheless, most of the information on these biomolecules is mainly available in the entire shells of crabs and shrimp. Therefore, this study, for the first time, reports the chemical composition of different parts of crab shells, namely carapaces, shoulders, claws, tips, and legs, as well as shrimp shells. This investigation aimed to facilitate the full utilization of shellfish waste and bridge the knowledge gap regarding the availability of various nutrients (amino acids, minerals, carotenoids, etc.) in different crab segments and shrimp shells.

## 2. Materials and Methods

### 2.1. Chemicals and Reagents

All solvents and chemicals used were of analytical or chromatographic grade and were purchased from Fisher Scientific Co. (Nepean, ON, Canada) and Sigma-Aldrich Ltd. (Oakville, ON, Canada).

### 2.2. Raw Materials (Shrimp and Crab Samples)

**Figure 1:**
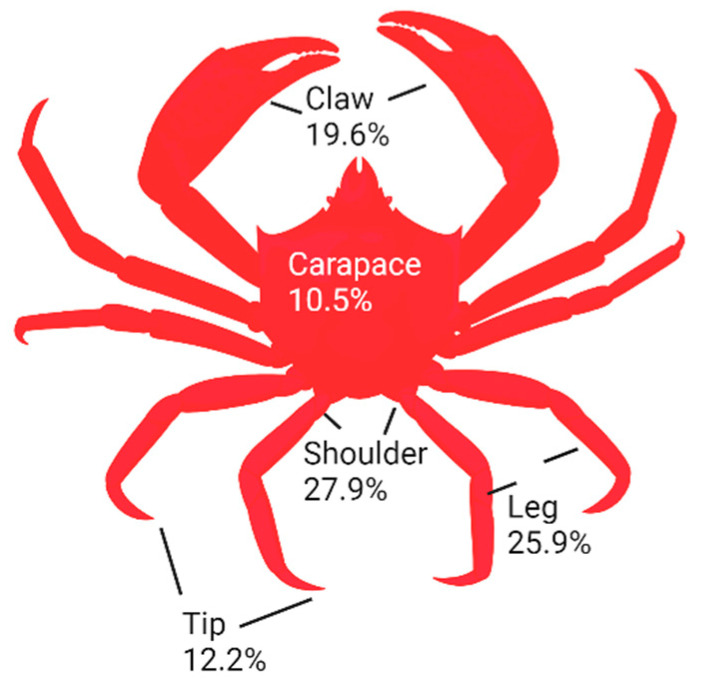
Percentage of different parts of crab in crab sections.

Shell wastes were prepared from the commercial processing of Northern shrimp (*Pandalus borealis*) and snow (queen) crab (*Chionoecetes opilio*) at Quinlan Brothers Fish Plant in Bay de Verde, Newfoundland. Fifty shellfish were used to obtain shells for this study. Different body parts (carapace, shoulder, claw, tip, and leg) of crabs were separated according to Figure 1. In particular, crab shoulders contained the higher number of shells (27.9%), followed by legs, claws, tips, and carapaces, which were determined on a fresh weight basis. After grinding, samples were vacuum-packed in plastic bags and stored at −20 °C for further analysis.

### 2.3. Proximate Analysis

The proximate composition of shell wastes (shrimp and crab body parts) was analyzed using the AOAC (2000) methods. The moisture content in shell wastes was measured by oven drying 2 g samples at 105 °C overnight or until a constant weight was reached. The protein content was determined by extracting a known amount of shell wastes with a 2.5% (*w*/*v*) KOH solution at 90 °C for 2 h according to the Kjeldahl method. The total lipid content was determined using the Bligh and Dyer [11] method. Moreover, ash content was measured by burning shell wastes (2 g) in a muffle furnace at 550 °C until the ash appeared white. The chitin content, on a dry weight basis, was determined by the demineralization of around 1 g of shells according to the method described by Shahidi and Synowiecki [12]. The deproteination was conducted with a 5% KOH solution for 2 h at 100 °C, followed by treatment with 20 mL of 5% HCl for 2 h at room temperature. The chitin was washed with deionized water until the pH reached 7.0, then rinsed with acetone, and finally oven-dried at 105 °C.

### 2.4. Amino Acid Analysis

The total amino acid content in shell waste proteins was extracted using a 2% KOH at 20 °C for 2 h, followed by precipitating with a 10% acetic acid at pH 4.5. The individual amino acids were determined after hydrolysis with 6 M HCl at 110 °C for 24 h. Individual amino acids were identified and quantified using a Beckman 121 MB amino acid analyzer. Tryptophan, methionine, and cysteine were identified based on the method described by Shahidi and Synowiecki [12]. Moreover, 80% ethanol was used to extract free amino acids and then deproteinized with 5-sulfosalicylic acid [13]. The individual free amino acid was determined, as discussed above.

### 2.5. Determination of Macro- and Micro-Elements

The content of macro- and micro-elements was determined according to the method described by Hossain et al. [14]. Briefly, shell waste (2 g) was mixed with 20 mL of HNO_3_ and heated on a hot plate at 90 °C overnight to remove the acid. The mixture was diluted in distilled water to make the volume 50 mL after cooling. The macro- and micro-elements were then analyzed using an inductively coupled plasma mass (ICP) spectrometer (iCAP7600 Duo, Thermo Scientific, ON, Canada). A calibration was performed using known standards for each mineral.

### 2.6. Determination of Carotenoid Content

The total carotenoid content of shell waste was examined based on the method stated by Onodenalore et al. [3]. Carotenoid fractions were fractioned using thin-layer chromatography (TLC). Carotenoid extracts in chloroform were spotted onto silica gel TLC plates and developed according to Shahidi and Synowiecki [12]. Each carotenoid component was then extracted three times with 3 mL of chloroform. After centrifugation, the mixture was diluted in 10 mL chloroform, and the absorbance was read using a spectrophotometer at 468 nm. Individual carotenoid content was quantified using authentic carotenoid standards in chloroform.

### 2.7. Statistical Analysis

The results presented in this study are based on processing discards collected from 50 shellfish. After mixing, three batches of discards were prepared for each body part, with each experiment analyzed in triplicate. The data were collected as the mean ± standard deviation of triplicate determination. Analysis of variance (ANOVA) was conducted, with mean values determined using Tukey’s studentized test at *p* < 0.05 using the statistical analysis system (SAS). The graphs were made using the Origin 9 software.

## 3. Results and Discussions

### 3.1. Proximate Composition

The proximate composition of fresh shrimp and crab shell wastes obtained from about 50 shellfish is presented in Table 1. The moisture content of shrimp shells was 73.4%, while that of crab shell wastes was 45.8–66.63%, depending on the crab shell body parts. In particular, crab claws and carapaces had the lowest and highest moisture content, respectively, among different crab sections. The protein content in shrimp shells was 10.73%, while different crab parts contained 7.63–12.5% protein, the shoulders being the highest, 12.5%. The lipid content of shrimp shell waste (1.5%) exceeded that of crab shell waste (0.3–0.93%) in different parts. However, the opposite was observed for total ash content, where shrimp shell waste was lower than crab shell waste. Specifically, crab claws contained a higher amount of ash (20.17%) compared to other body parts. On a dry weight basis, shrimp shells, crab carapaces, shoulders, claws, tips, and legs contained about 42, 75, 69, 77, 75, and 82% chitin, respectively, suggesting that legs and claws could be graded as rich reservoirs of chitin.

The result from the proximate composition is comparable with those of Burke and Kerton [5], Rødde et al. [4], and Heu et al. [13] for the same species of crab (*Chinoecetes opilio*) and shrimp (*Pandalus borealis*). Nevertheless, the differences in the results may originate from the processing methods (e.g., cooking and drying) employed for shrimp and crab as well as techniques used to determine the composition. In particular, the proximate composition was evaluated from raw/fresh shrimp and crab shell wastes, while most of the studies used dry matter to examine the composition. Tremblay et al. [6] stated that cooking of shellfish releases proteins, peptides, and amino acids. Additionally, the proximate composition of different body parts of snow crab wastes is not well-detailed to compare our results.

### 3.2. Amino Acid Composition

Amino acids of shrimp shell waste and crab shoulders were determined since this particular body part of the crab had a comparatively higher protein content. The contents of total and free amino acids are shown in Table 2 and Table 3, respectively.

Results indicate that shrimp shell proteins are well-balanced in their essential amino acid composition, and as such, they can be used as an excellent source of protein-rich value-added food ingredients or in animal feed and aquaculture industries. In fact, for most amino acids, the contents were fairly close to that of milk. In contrast, crab shoulder protein contained much lower levels of amino acids, but alanine, arginine, aspartic acid, glutamic acid, glycine, proline, valine, and taurine were abundant, contributing about 70% of total amino acids. Thus, to prepare value-added products, crab shell wastes may need to be complemented with other proteins. Similar trends were also observed in shrimp shell waste, where these amino acids were also predominant (around 70% of total amino acids), promoting the growth, development, and health of shellfish. Among all amino acids, arginine was found in a higher quantity in the crab shoulder (12.92 g/100 g), whereas glycine was abundant in shrimp shells (12.92 g/100 g) (Table 2). In contrast, free amino acids, mainly alanine, arginine, glycine, proline, sarcosine, and taurine, were abundant in both crab shoulder and shrimp, contributing about 66 and 68% of total free amino acids, respectively (Table 3). Notably, arginine was the most predominant free amino acid in the crab shoulder (17.13 mg/100 g shells), whereas glycine was abundant in shrimp shells (32.51 mg/100 g shells). Arginine is a precursor for creatine and plays an important role in polyamine synthesis, producing nitric oxide and innate immune responses, while glycine modulates the antioxidant system of shellfish, improving the immune system [15]. Interestingly, a small amount of asparagine and glutamine was detected in the free amino acids of both samples.

The reported amino acid contents are aligned with those reported by Naczk et al. [16], Tremblay et al. [6], and Heu et al. [13] for proteins obtained from green crab (*Carcinus maenas*), snow crab (*Chionoecetes opilio*), and Northern shrimp (*Pandalus borealis*). In particular, glycine was identified in shrimp (*Parapenaeopsis stylifera*) shells harvested from the Indian Ocean [17], while arginine and taurine were the major amino acids in snow crabs harvested from the Atlantic Ocean [6]. Free amino acids are crucial in contributing to the flavor and taste of foods, including seafood. The primary taste active amino acids in crabs are glutamic acid, arginine, and glycine (Table 3), and the absence of any of these compounds is expected to result in a decrease in sweetness and the umami taste. For example, the major non-volatile taste-active compounds in crabs are mainly glutamic acid, aspartic acid, glycine, serine, alanine, histidine, lysine, and valine, among others [18]. Among them, glycine and alanine are the primary contributors to the sweet taste, whereas glutamic acid and aspartic acid are responsible for the strong umami [19]. Therefore, the levels and relative proportions of amino acids in shellfish can significantly impact both consumers and seafood producers.

### 3.3. Micro- and Macro-Element Analysis

**Figure 2:**
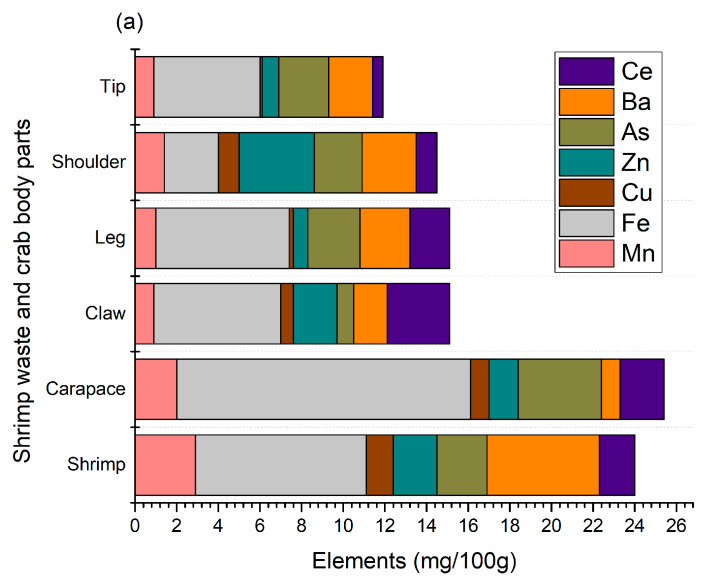

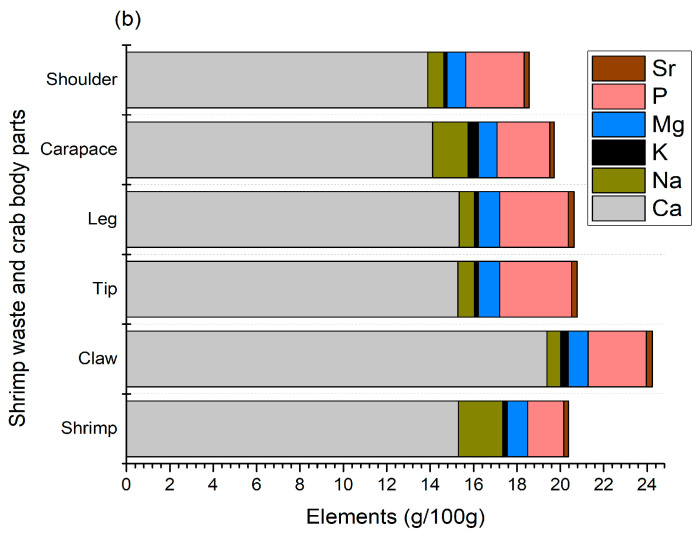
Micro- (**a**) and macro- (**b**) elements of shrimp and crab shell wastes.

Crustaceans need a lot of minerals, primarily calcium and magnesium, for their shells. They are known for their mineralized chitinous exoskeleton, which is strengthened with calcium salts. Without enough minerals, they cannot form a new undercarapace for molting, leading to molting issues [20]. The main purpose of this mineralization is to increase the mechanical strength of their skeleton. Thus, this study determined the minerals in shellfish waste, as shown in Figure 2.

A total of seven micro-elements (Mn, Fe, Cu, Zn, As, Ba, and Ce) were identified from shellfish waste, where iron is predominant and present at 2.6–14.1 mg/100 g (Figure 2a). Crab carapaces contained the highest amount (*p* < 0.05) of iron compared to other crab sections, as well as shrimp shell wastes. AlFaris et al. [9] found a similar trend in shrimp (*Penaeus semisulcatus*), where iron was the predominant element compared to other minerals such as zinc and copper. Moreover, similar results were found in Northern shrimp (*Pandalus borealis*) harvested from the Barents Sea, and the levels of iron, barium, and copper were 3.07–5.93, 1.73–2.48, and 1.03–1.88 mg/100 g, respectively [4]. In our study, these levels were 2.6–14.1, 0.9–5.4, and 0.1–1.3, respectively, and the variolation could be related to the geographical location, type of feed, harvesting season, and extraction method. Shrimp shells were good sources of manganese, while crab sections, mainly shoulders, contained zinc. The presence of arsenic is also found in shellfish waste, mainly crab carapaces, and this could be associated with the distinct age, feed, and water salinity of their habitat. Shellfish feed mainly on algal materials and concentrate arsenic compounds. However, because organic arsenics are relatively low in toxicity, there is not much concern about arsenic levels [21,22]. Aside from this, the presence of Mn, Zn, and Fe is essential for maintaining certain biological functions, including blood sugar regulation and bone growth maintenance [23]. Usually, Fe is the most abundant element in shellfish waste and is mainly accumulated from feed, groundwater contamination from mining, and industrial effluents, among others. Fe is essential for the activation of coenzymes, binding proteins, and other metabolic functions for humans [24]. Nonetheless, the levels of these micro-elements, mainly Zn and Mn, were slightly higher than their allowable levels set by FAO/WHO (1989), which are aligned with the findings of Baki et al. [25]. The concentrations mostly depend on specific tissue/ species harvested from different geographical locations.

The profile of macro-elements identified from shrimp and crab shell waste is shown in Figure 2b. A total of six macro-elements, namely calcium, sodium, potassium, magnesium, phosphorus, and strontium, were identified with a calcium constitution of over 60%. Meanwhile, phosphorus and sodium were the second and third highest macro-elements, respectively. High levels of calcium and phosphorus are expected in crustacean shells since they are the major elements responsible for the formation and strengthening of their hard shells. In terms of crab body parts, crab claws contained a higher number of macro-elements, mainly calcium (19.39 g/100 g), which is also reflected in the total ash content, as shown in Table 1. The results of this study are in agreement with those of AlFaris et al. [9], Heu et al. [13], and Rødde et al. [4], who found that shrimps and their shells are a good source of calcium, magnesium, phosphorus, sodium, and potassium. In particular, AlFaris et al. [9] identified strontium in northern shrimp (*Pandalus borealis*) shells, thus supporting our findings. On the other hand, Ahmed [26] suggested that crab shells have higher calcium and magnesium contents than crab meat. Therefore, shell waste minerals, mainly calcium, phosphorus, and iron, can be utilized as feed supplements or other value-added products to contribute to better bone health and overall growth.

### 3.4. Carotenoids

**Figure 3:**
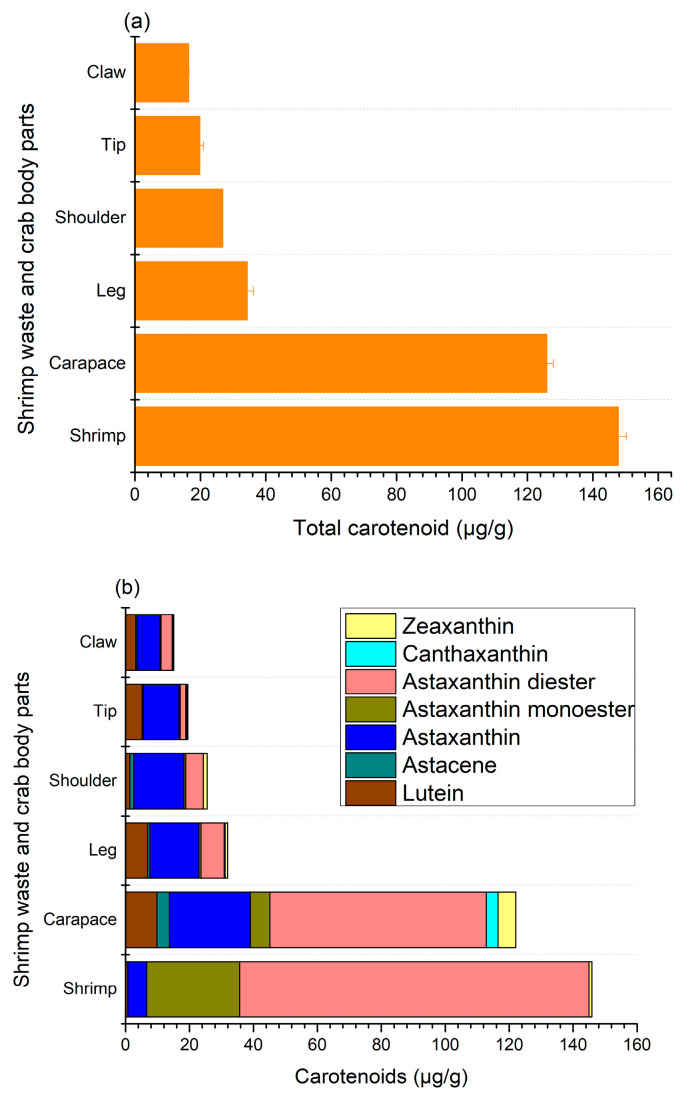
Total (**a**) and individual (**b**) carotenoid contents in shrimp and crab shell wastes.

In shellfish, body color is determined by the pigments found in the main layer of their exoskeleton and the subepidermal chromatophores. The most prevalent pigment in shellfish is carotenoids, mainly astaxanthin, along with canthaxanthin, zeaxanthin, lutein, and astacene [27]. The total carotenoid and individual carotenoid contents observed in this study are given in Figure 3, where shrimp shells (147.7 ± 2.5 μg/g) and crab carapaces (125.9 ± 2 μg/g) had the highest content of pigments (Figure 3a).

The content of carotenoids in other crab sections varied from 16.4 ± 0.1 to 34.3 ± 2.0 μg/g, where claws and tips had the lowest levels of carotenoids. The major components of carotenoids for shrimp shells and crab carapaces were mono- and di-esters of astaxanthin Figure 3b, mainly the diester form at 109.22 ± 2.8 and 67.66 ± 2.65 μg/g, respectively. Nonetheless, astaxanthin esters accounted for less than a quarter of the total carotenoid levels of other body parts of crab shells. Astaxanthins are usually found in crustaceans as free or esterified (mono- and di-esterified) forms with different fatty acids, such as palmitic, oleic, stearic, or linoleic acids, and are principally responsible for the color of shrimps and crabs. In addition, shrimp and crab shell wastes also contained free astaxanthin as well as a small amount of zeaxanthin and lutein, while astacene and canthaxanthin were only detected in crab parts, with the exception of crab shoulders, where no canthaxanthin was found. In particular, the contents of free astaxanthin in shrimp shells and crab carapaces, shoulders, claws, tips, and legs were 5.88 ± 0.6, 25.3 ± 1.95, 15.69 ± 1.1, 7.21 ± 0.9, 11.18 ± 1.2, and 15.34 ± 1.25 μg/g, respectively. These findings demonstrate that astaxanthin and its esters are major carotenoid components in shrimp and crab shells, which is in agreement with previous reports on various species of crab and shrimp [4,5,12,16,17,28,29]. In particular, Rødde et al. [4] and Shahidi and Synowiecki [12] found a similar trend in the same species of shrimp (*Pandalus borealis*), where the main form of astaxanthin was diester, followed by monoester and free astaxanthin. Similarly, astaxanthin diester was identified as the major carotenoid present in green crab (*Carcinus maenas*), snow crab (*Chinoecetes opilio*), and blue crab (*Portunus segnis*) shells, along with astaxanthin and its monoester, as well as zeaxanthin, astacene, canthaxanthin, and lutein [12,16,28]. It had previously been proposed that esters may serve a storage function and could facilitate the incorporation of astaxanthin into crustacyanin, a carotenoprotein, thereby influencing shell colors [29]. In crustaceans, astaxanthin is found in carotenoproteins, displaying purple, yellow, and blue colors.

**Figure 4:**
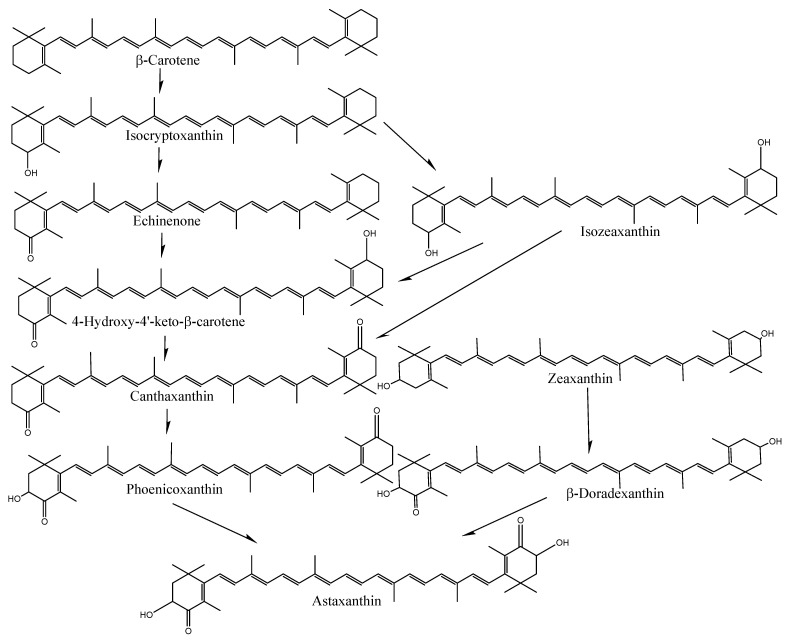
Conversion of carotenoids in shrimp-like fish.

The species- and tissue-specific differences in the accumulation of carotenoids in crabs and shrimp indicate distinct uptake and deposition mechanisms. In particular, the bioavailability and bioconversion of carotenoids are mainly affected by various factors, including the type of carotenoid and its molecular linkage, the presence of matrix effectors that influence absorption and bioconversion, the content of carotenoids ingested in a meal, genetic factors, nutritional status, host-related matters, and the complex interactions among these elements [29,30]. However, the exact reasons for varying carotenoid content among tissues or species are still unknown. Crustaceans cannot synthesize astaxanthin on their own; only plants and protists, such as algae, bacteria, and fungi, can produce carotenoids. As a result, astaxanthin needs to be present in their natural environment or provided in their diet to fulfill their metabolic nutritional needs. Astaxanthin is produced by microalgae or phytoplankton at the base of the food chain in natural aquatic environments. These microalgae are eaten by insects, zooplankton, or crustaceans, which accumulate astaxanthin. Thus, crustaceans can synthesize astaxanthin from β-carotene and zeaxanthin through a series of oxidation and hydroxylation reactions (Figure 4) or store astaxanthin directly by having other foods that already performed the bioconversion [2].

## 4. Conclusions

This study is the first to examine the proximate composition and the specific amino acids, minerals, and carotenoids present in various parts of snow crabs and shrimp shells. The findings reveal that shrimp proteins are of very high quality due to their amino acid composition, while snow crab shells show varying amino acid content depending on the specific part. In addition, free amino acids are expected to have a major influence on aroma and taste. Moreover, seven micro-elements and six macro-elements were identified and quantified; among them, calcium and iron were most abundant in the crab carapaces. Additionally, both shrimp shells and crab carapaces were rich in carotenoids, mainly astaxanthin and its esters, as well as zeaxanthin, astacene, canthaxanthin, and lutein. Therefore, shellfish wastes could serve as a useful source of protein, chitin, minerals, and carotenoids in food/ feed formulations. By isolating and determining the specific nutrients present in each part, one can more efficiently target their application based on their unique compositions. For example, the high-quality shrimp proteins could be optimized for human consumption, while the mineral-rich crab carapaces, abundant in calcium and iron, could be used for dietary supplements or feed formulations. This separation could lead to more tailored uses of shellfish waste, enhancing resource efficiency in a circular economy and reducing environmental impact.

## Figures and Tables

**Table 1:** Proximate composition of shell waste from shrimp and snow crab.

%	Crab (*Chionoecetes opilio*)					Shrimp (*Padalus borealis*)
	Carapace	Shoulder	Claw	Tip	Leg	Shell Waste
Moisture	66.63 ± 2.40 ^b^	59.47 ± 1.34 ^c^	45.80 ± 0.66 ^f^	51.03 ± 1.81 ^d^	48.67 ± 0.21 ^e^	73.40 ± 2.08 ^a^
Proteins	9.40 ± 0.70 ^b^	12.50 ± 0.20 ^a^	8.90 ± 0.30 ^c^	10.03 ± 1.37 ^bc^	7.63 ± 0.35 ^d^	10.73 ± 1.37 ^b^
Lipids	0.93 ± 0.10 ^b^	0.30 ± 0.10 ^c^	0.43 ± 0.15 ^c^	0.37 ± 0.06 ^c^	0.40 ± 0.10 ^c^	1.50 ± 0.19 ^a^
Ash	17.93 ± 1.33 ^b^	15.23 ± 1.31 ^c^	20.17 ± 0.31 ^a^	15.97 ± 0.31 ^c^	17.60 ± 0.87 ^b^	7.83 ± 0.23 ^d^
Chitin *	75.01 ± 2.10 ^b^	69.04 ± 1.48 ^c^	77.02 ± 2.08 ^b^	75.04 ± 1.75 ^b^	82.03 ± 1.98 ^a^	42.02 ± 0.52 ^d^

* Percentage on a dry basis. Results represent the mean of three determinations ± standard deviation. Values in the same row with different lowercase letters are significantly different (*p* < 0.05).

**Table 2:** Total amino acid content in proteins extracted from crab shoulder and shrimp shell waste.

Amino Acid	Crab Shoulder	Shrimp Shell
	g/100 g Protein	g/100 g Protein
Alanine	6.48 ± 0.01	6.62 ± 0.10
α-Aminoadipic acid	0.10 ± 0.01	-
α-Aminobutyric acid	0.05 ± 0.00	-
δ-Aminobutyric acid	0.02 ± 0.00	-
Arginine	12.92 ± 0.09	8.22 ± 0.05
Aspartic acid	6.17 ± 0.15	5.92 ± 0.24
Citrulline	0.01 ± 0.00	-
Cystathionine	0.02 ± 0.00	-
Cystine	0.64 ± 0.01	0.10 ± 0.02
Glutamic acid	7.47 ± 0.07	8.19 ± 0.22
Glycine	11.37 ± 0.05	12.90 ± 0.27
Histidine	1.81 ± 0.08	1.35 ± 0.00
Hydroxyproline	0.03 ± 0.01	0.05 ± 0.04
Isoleucine	2.81 ± 0.00	3.13 ± 0.01
Leucine	4.39 ± 0.03	4.38 ± 0.09
Lysine	2.79 ± 0.01	3.98 ± 0.07
Methionine	1.62 ± 0.01	0.98 ± 0.01
1-Methylhistidine	0.01 ± 0.00	-
3-Methylhistidine	0.12 ± 0.01	-
Ornithine	0.14 ± 0.01	0.76 ± 0.01
Phenylalanine	3.15 ± 0.03	2.95 ± 0.05
Proline	6.57 ± 0.02	9.56 ± 0.4
Serine	2.67 ± 0.03	3.21 ± 0.00
Taurine	8.43 ± 0.02	7.56 ± 0.01
Threonine	2.75 ± 0.08	2.68 ± 0.02
Tryptophan	0.39 ± 0.00	0.46 ± 0.01
Tyrosine	2.28 ± 0.02	2.01 ± 0.06
Valine	5.32 ± 0.02	4.60 ± 0.26
Sarcosine	2.86 ± 0.00	4.66 ± 0.10

**Table 3:** Free amino acid composition of crab shoulder and shrimp shell waste.

Amino Acid	Crab Shoulder		Shrimp Shell	
	mg/g Protein	mg/100 g Shells	mg/g Protein	mg/100 g Shells
Alanine	16.56 ± 0.01	6.64 ± 0.09	26.85 ± 0.75	9.56 ± 0.26
α-Aminoadipic acid	0.57 ± 0.01	0.20 ± 0.00	0.56 ± 0.00	0.20 ± 0.00
α-Aminobutyric acid	0.25 ± 0.01	0.10 ± 0.00	-	-
δ-Aminobutyric acid	0.16 ± 0.00	0.06 ± 0.00	0.24 ± 0.00	0.08 ± 0.0
Arginine	42.73 ± 0.27	17.13 ± 0.11	40.94 ± 1.37	14.57 ± 0.48
Aspartic acid	1.77 ± 0.01	0.71 ± 0.00	6.81 ± 0.39	2.42 ± 0.14
Asparagine	0.37 ± 0.00	0.15 ± 0.00	1.11 ± 0.01	0.40 ± 0.00
Citrulline	0.35 ± 0.01	0.14 ± 0.00	0.41 ± 0.13	0.15 ± 0.05
Cystathionine	0.45 ± 0.01	0.18 ± 0.00	0.83 ± 0.03	0.30 ± 0.01
Cystine	0.65 ± 0.00	0.26 ± 0.00	0.23 ± 0.00	0.08 ± 0.00
Glutamic acid	4.25 ± 0.10	1.7 ± 0.04	12.39 ± 0.02	4.41 ± 0.01
Glutamine	2.37 ± 0.05	0.95 ± 0.02	6.67 ± 0.01	2.34 ± 0.00
Glycine	16.95 ± 0.17	6.8 ± 0.07	92.62 ± 4.21	32.51 ± 1.5
Histidine	3.15 ± 0.26	1.26 ± 0.10	2.08 ± 0.02	0.73 ± 0.01
Hydroxyproline	0.65 ± 0.02	0.26 ± 0.01	-	-
Isoleucine	5.93 ± 0.17	2.38 ± 0.07	8.52 ± 0.05	2.99 ± 0.02
Leucine	8.14 ± 0.18	3.26 ± 0.07	12.02 ± 0.38	4.22 ± 0.12
Lysine	3.83 ± 0.08	1.53 ± 0.03	10.85 ± 0.38	3.81 ± 0.13
Methionine	6.83 ± 0.10	2.74 ± 0.04	1.4 ± 0.00	0.50 ± 0.00
1-Methylhistidine	0.4 ± 0.00	0.16 ± 0.00	-	-
3-Methylhistidine	0.81 ± 0.0	0.32 ± 0.00	-	-
Ornithine	0.26 ± 0.00	0.1 ± 0.00	5.11 ± 0.22	1.8 ± 0.07
Phenylalanine	8.22 ± 0.26	3.3 ± 0.10	7.98 ± 0.24	2.8 ± 0.08
Proline	23.25 ± 0.02	9.32 ± 0.10	48.64 ± 1.91	17.0.7 ± 0.68
Serine	2.55 ± 0.00	1.02 ± 0.00	6.3 ± 0.24	2.21 ± 0.08
Taurine	20.98 ± 0.03	8.41 ± 0.02	39.06 ± 0.05	13.71 ± 0.02
Threonine	1.67 ± 0.00	0.67 ± 0.00	6.25 ± 0.33	2.19 ± 0.11
Tryptophan	1.6 ± 0.01	0.64 ± 0.00	2.0 ± 0.22	0.70 ± 0.01
Tyrosine	6.55 ± 0.21	2.62 ± 0.08	7.41 ± 0.23	2.6 ± 0.08
Valine	7.69 ± 0.08	3.08 ± 0.04	7.85 ± 0.48	2.75 ± 0.016
Sarcosine	16.58 ± 0.12	6.64 ± 0.05	30.14 ± 1.13	10.58 ± 0.40

## Data Availability

The original contributions presented in the study are included in the article, further inquiries can be directed to the corresponding author.
